# A Novel Purification Procedure for Active Recombinant Human DPP4 and the Inability of DPP4 to Bind SARS-CoV-2

**DOI:** 10.3390/molecules25225392

**Published:** 2020-11-18

**Authors:** Cecy R Xi, Arianna Di Fazio, Naveed Ahmed Nadvi, Karishma Patel, Michelle Sui Wen Xiang, Hui Emma Zhang, Chandrika Deshpande, Jason K K Low, Xiaonan Trixie Wang, Yiqian Chen, Christopher L D McMillan, Ariel Isaacs, Brenna Osborne, Ana Júlia Vieira de Ribeiro, Geoffrey W McCaughan, Joel P Mackay, W Bret Church, Mark D Gorrell

**Affiliations:** 1 Centenary Institute, Faculty of Medicine and Health, The University of Sydney, Sydney, NSW 2006, Australia; c.xi@centenary.org.au (C.R.X.); arianna.difazio@path.ox.ac.uk (A.D.F.); naveed.nadvi@sydney.edu.au (N.A.N.); m.xiang@centenary.org.au (M.S.W.X.); e.zhang@centenary.org.au (H.E.Z.); t.wang@centenary.org.au (X.T.W.); yiqian.chen@hudson.org.au (Y.C.); brenna@sund.ku.dk (B.O.); anajulia.vribeiro@gmail.com (A.J.V.d.R.); g.mccaughan@centenary.org.au (G.W.M.); 2 Research Portfolio Core Research Facilities, The University of Sydney, Sydney, NSW 2006, Australia; 3 Faculty of Science, School of Life and Environmental Sciences, The University of Sydney, Sydney, NSW 2006, Australia; kpat7951@uni.sydney.edu.au (K.P.); chandrika.deshpande@sydney.edu.au (C.D.); jason.low@sydney.edu.au (J.K.K.L.); 4 Drug Discovery, Sydney Analytical, Core Research Facilities, The University of Sydney, Sydney, NSW 2006, Australia; joel.mackay@sydney.edu.au; 5 School of Chemistry and Molecular Biosciences, The University of Queensland, St Lucia, QLD 4072, Australia; c.mcmillan1@uq.edu.au (C.L.D.M.); ariel.isaacs@uq.net.au (A.I.); 6 AW Morrow GE & Liver Centre, Royal Prince Alfred Hospital, Camperdown, NSW 2050, Australia; 7 Faculty of Medicine and Health, School of Pharmacy, The University of Sydney, Sydney, NSW 2006, Australia; bret.church@sydney.edu.au

**Keywords:** recombinant protein, protease, DPP4, Covid-19

## Abstract

Proteases catalyse irreversible posttranslational modifications that often alter a biological function of the substrate. The protease dipeptidyl peptidase 4 (DPP4) is a pharmacological target in type 2 diabetes therapy primarily because it inactivates glucagon-like protein-1. DPP4 also has roles in steatosis, insulin resistance, cancers and inflammatory and fibrotic diseases. In addition, DPP4 binds to the spike protein of the MERS virus, causing it to be the human cell surface receptor for that virus. DPP4 has been identified as a potential binding target of SARS-CoV-2 spike protein, so this question requires experimental investigation. Understanding protein structure and function requires reliable protocols for production and purification. We developed such strategies for baculovirus generated soluble recombinant human DPP4 (residues 29–766) produced in insect cells. Purification used differential ammonium sulphate precipitation, hydrophobic interaction chromatography, dye affinity chromatography in series with immobilised metal affinity chromatography, and ion-exchange chromatography. The binding affinities of DPP4 to the SARS-CoV-2 full-length spike protein and its receptor-binding domain (RBD) were measured using surface plasmon resonance and ELISA. This optimised DPP4 purification procedure yielded 1 to 1.8 mg of pure fully active soluble DPP4 protein per litre of insect cell culture with specific activity >30 U/mg, indicative of high purity. No specific binding between DPP4 and CoV-2 spike protein was detected by surface plasmon resonance or ELISA. In summary, a procedure for high purity high yield soluble human DPP4 was achieved and used to show that, unlike MERS, SARS-CoV-2 does not bind human DPP4.

## 1. Introduction

Dipeptidyl peptidase 4 (DPP4)**,** also known as CD26 and adenosine deaminase binding protein (ADAbp), is a 110 kDa type II transmembrane glycoprotein belonging to the DPP4 gene family of serine proteases. DPP4 is widely expressed on endothelial, epithelial and immune cells in mammalian tissues and has multifunctional roles in metabolism, immunology, endocrinology, fibrosis and cancer [1,2,3,4,5,6]. DPP4 is often cleaved from cell surfaces to be released into extracellular spaces as an enzymatically active, soluble form that has intact protein-protein binding activities [7,8,9,10]. Soluble DPP4 has been associated with a variety of diseases as a potential biomarker and is largely derived from damaged hepatocytes and activated lymphocytes [3,10,11]. DPP4 expression can be stimulated by hypoxia [12].

The soluble form of DPP4 is composed of an *α*/*β*-hydrolase domain and an eight-blade *β*-propeller domain with an active site located at the interphase of the two domains [9,13,14]. DPP4 preferentially cleaves after proline or alanine in the penultimate position from the *N*-terminus of polypeptides. DPP4 cleavage of the incretin peptides, glucagon-like peptide-1 (GLP-1) and gastric inhibitory polypeptide (GIP), have led to the development of DPP4 selective inhibitors as a successful type 2 diabetes mellitus (T2DM) therapy [4,15,16,17,18]. The outer surface of the propeller domain of DPP4 contains binding sites for other proteins, most notably the non-catalytic binding with human adenosine deaminase (ADA) [7,9,19,20]. DPP4 association with ADA has a co-stimulatory role in human T-cell activation [10]. The immunoregulatory and cardiovascular roles of DPP4 may be important in viral infections [10,21,22,23].

The MERS-CoV and SARS-CoV-2 epidemics arose about eight years apart. These viruses have a 50% genetic similarity [24]. MERS-CoV arose in 2012, and 2519 infections and 866 deaths have been reported [25], whereas SARS-CoV-2 caused more than 500,000 deaths within seven months. Infection by coronavirus is mediated by the binding of the surface spike glycoprotein to a host cell receptor via the receptor-binding domain (RBD) in the S1 subunit of the spike. MERS-CoV coronavirus infection is mediated by the S1 spike glycoprotein binding to DPP4 on a site that overlaps with the ADA binding site on the β-propeller domain of DPP4 [26,27]. Recent modelling of the homotrimer structure of SARS-CoV-2 spike has predicted a similar ability to bind to DPP4 [28]. Angiotensin-converting enzyme 2 (ACE2) has been experimentally validated as a high-affinity entry receptor for SARS-CoV-2 [29,30,31,32,33]. However, the potential binding interaction between DPP4 and SARS-CoV-2 is yet to be directly evaluated.

Molecular investigations on protein structure, function, substrates and binding partners require reliable methods to produce and purify DPP4. The most common approaches for expressing enzymatically active recombinant human DPP4 uses baculovirus-infected insect cells [13,14,34,35], and less commonly mammalian and yeast cells [10,36]. Baculovirus-insect expression systems have many advantages over mammalian and yeast expression systems, including the ease of use, adequate glycosylation, appropriate codon usage and potentially greater expression [37,38]. Although glycosylation of recombinant proteins in insect cells is less complex than the native human glycoproteins, it has produced stable and active soluble recombinant human DPP4 proteins [13].

Most commonly, recombinant soluble human DPP4 has been purified from insect cell cultures as an affinity-tagged protein using only immobilised metal affinity chromatography (IMAC) and size-exclusion chromatography [13,34,35,39]. Monomeric DPP4 is inactive, whereas dimeric and tetrameric forms are active, so we explored methods that avoided separation by size. We have shown that DPP4 activity is intolerant of *C*-terminal truncation, but tolerates some *N*-terminal truncations [40], so the affinity tag was attached to the *C*-terminus to maximise capture of active DPP4 by an IMAC resin. Here, we report an optimised 4-step purification strategy for His_6_-tagged soluble recombinant human DPP4 protein from Sf9 insect cells that achieved high purity. The purified DPP4 was used to measure the extent of binding with SARS-CoV-2 spike using ELISA and surface plasmon resonance (SPR).

## 2. Results

### 2.1. The Expression and Purification of DPP4

A His_6_-tagged soluble form of human DPP4 (residues 29 to 766) was generated in a pMelbac baculovirus vector as we have described previously [35]. When stored in sterile conditions below 10 °C, with glycerol to prevent freezing, DPP4 activity in cell culture supernatants did not detectably diminish within seven years. This form of soluble DPP4 is stable and fully active in both enzyme activity and ADA binding [10,41]. For this study, the recombinant virus was further cloned to select for increased DPP4 production. Enzyme activity is dependent on the structural integrity of these proteins [3,40,42]. Hence, expression levels were assessed by measuring the enzymatic activity of cell cultures. DPP4 expression was optimised by varying the quantity of virus for infection and the time to harvest. The time course of enzymatic activity after infection showed that robust enzyme expression was achieved 7 days post-infection. Overall, a 1 L suspension of Sf9 cells (1.2–2.2 × 10^6^ cells/mL) was infected at a multiplicity of infection (MOI) of 1 and harvested 7 days post-infection for large-scale DPP4 protein expression.

**Figure 1:**
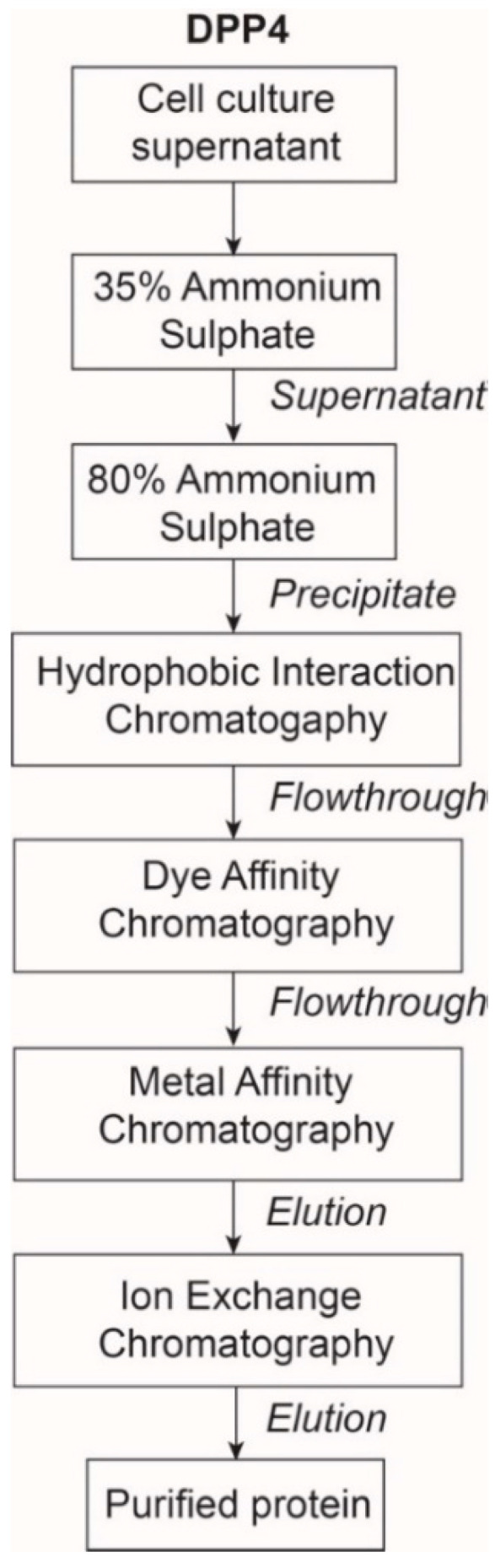
Overview of DPP4 purification workflow.

DPP4 protein was purified in four-steps: Ammonium sulphate (AS) precipitation, hydrophobic interaction chromatography (HIC), dye affinity chromatography in series with IMAC, and ion-exchange chromatography (IEX) (Figure 1). The purification process was monitored by measuring specific activity, with the goal of achieving >20 U/mg because above 20 U/mg has been established as the specific activity of highly purified DPP4 [8].

**Figure 2:**
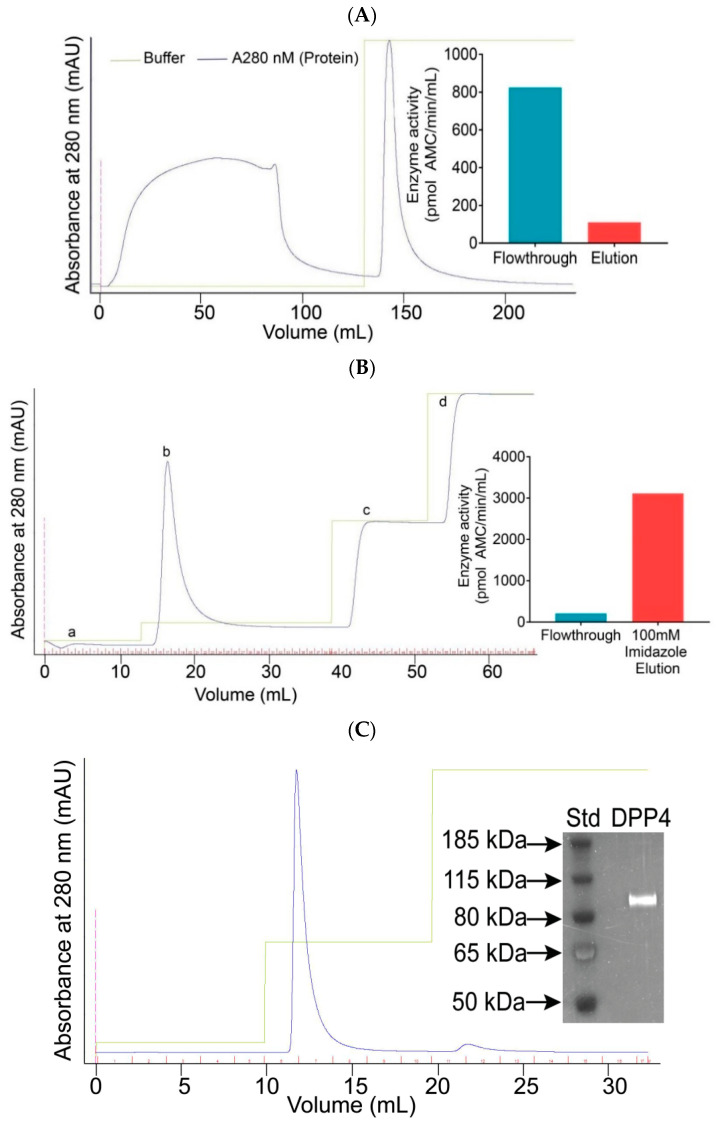
Elution profiles of DPP4 chromatography. (**A**) Chromatogram from Phenyl Sepharose that was equilibrated with 12% ammonium sulphate (AS) in 10 mM Tris-HCl pH 7.6 and eluted with 0% AS in 10 mM Tris-HCl pH 7.6 buffer. Inset: Fibroblast activation protein (FAP) activity. (**B**) Chromatogram from Nickel Sepharose, which was equilibrated with 20 mM imidazole in 200 mM NaCl, 10 mM Tris-HCl, pH 7.6 and eluted with an increasing concentration gradient of imidazole at 30 mM (a), 100 mM (b), 500 mM (c), 1000 mM (d) in 10 mM Tris-HCl pH 7.6 buffer. Inset: FAP activity. (**C**) Chromatogram from DEAE Sepharose that was equilibrated with 10 mM Tris-HCl pH 7.6 and eluted with 200 mM NaCl in 10 mM Tris-HCl pH 7.6 buffer. Inset: sodium dodecyl sulphate polyacrylamide gel electrophoresis (SDS–PAGE; 4–12% Bis-Tris gel) of the resulting purified soluble DPP4, stained with Sypro ruby Protein was measured by optical density at 280 nm in these chromatograms.

For a preliminary separation of DPP4 from contaminants in the cell culture supernatant, precipitation at 35% AS saturation followed by 80% AS saturation was performed (Figure S1) [35]. This step improved enzyme purity 7-fold with a 59% yield (Table 1). Next, to exploit the presence of AS and remove some contaminating hydrophobic proteins, HIC using Phenyl Sepharose with 12% AS was performed, similar to a previous study [43]. HIC produced two major elution peaks: The first peak represents unbound non-hydrophobic soluble DPP4 in the flowthrough and the second peak represents contaminants eluted from the Phenyl Sepharose column with a 0% AS buffer (Figure 2A).

Blue Sepharose dye affinity chromatography binds to albumin and some other contaminants [44] and did not bind to DPP4. DPP4 protein was then captured from the solution by IMAC that was directly attached downstream in series with the Blue Sepharose column. The IMAC technique utilises the affinity of exposed poly-histidine towards charged transition metals, such as Ni^2+^, immobilised on a chelated chromatography resin. An increasing concentration gradient of imidazole at 30 mM, 100 mM, 500 mM and 1000 mM revealed that DPP4 eluted from the Nickel Sepharose column at 100 mM imidazole, in a single sharp peak (Figure 2B). Imidazole at 30 mM eluted little or no DPP4. Therefore, subsequent purification runs loaded the IMAC in 30 mM imidazole and eluted DPP4 with 100 mM imidazole. IMAC was the central and most effective purification step, increasing the DPP4 protein purity to >40 U/mg specific activity with a 46% yield and losing only 16% of the DPP4 that was loaded onto IMAC (Table 1). IMAC achieved greater than 2000-fold purification compared to culture supernatant.

To concentrate the DPP4, the DPP4-enriched fractions from IMAC were combined for IEX on DEAE Sepharose. As IEX was performed at pH 7.6, DPP4 was expected to be negatively charged (pI is approximately 6) and bind to the positively charged DEAE Sepharose. Bound DPP4 proteins were eluted with 200 mM NaCl, seen as a sharp peak on the chromatogram (Figure 2C). A second smaller peak was observed, which did not contain DPP4 activity and may represent protein contaminants that bind more tightly to the IEX resin. This final IEX step concentrated the DPP4, while maintaining purity (judged by specific activity); whereas, it decreased yield to 29% and caused a 35% loss of DPP4 (Table 1).

The purified DPP4 was analysed by SDS-PAGE with Sypro ruby and Colloidal blue stains. Heating samples for PAGE monomerises DPP4 and thereby simplifies the interpretation of gels. A single protein band was obtained near the expected molecular mass of monomeric DPP4 (Figure 2C, Figure S2). The calculated monomeric size of DPP4 (29–766) is 88.3 kDa and glycosylation occurring in this expression system is limited [14]. When stored in sterile conditions, the purified DPP4 was very stable at 0 °C to 30 °C, and when 10% glycerol was added activity was undiminished for more than a year in storage below 0 °C. For example, two activity measurements made 15 months apart were 11.7 and 11.2 U/mL.

The mean specific activity of the purified DPP4 enzyme obtained from five separate purification runs was 36 U/mg (Table 2). This optimised protocol yielded, on average 1.4 mg of DPP4 protein per litre of suspension cell culture (Table 2).

### 2.2. DPP4, MERS-CoV and SARS-CoV-2

**Figure 3:**
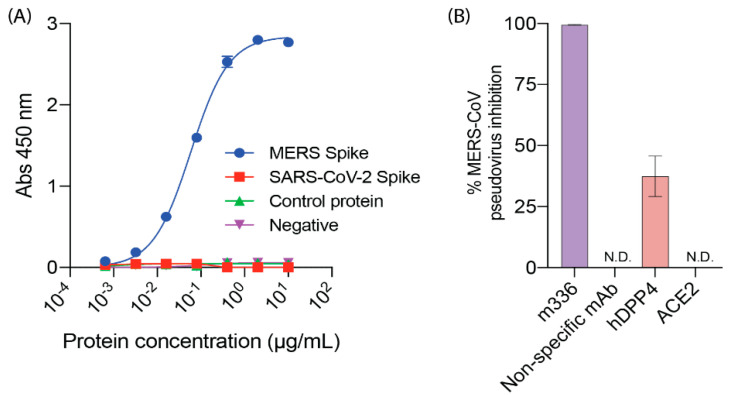
ELISA and virus neutralisation assays. (**A**) Purified DPP4 protein was used to capture MERS-CoV spike clamp, SARS-CoV-2 spike clamp or a control clamped protein. Clamp-stabilised proteins were detected using a clamp-specific mAb HIV1281. Data shown represent means of duplicate values. (**B**) DPP4, ACE2, a MERS-specific mAb m336 or a non-specific mAb C05 were used at 20 µg/mL and incubated with MERS-CoV pseudovirus. Percent MERS-CoV inhibition is the percentage reduction in luciferase signal (RLU) compared to virus-only control. N.D. indicates no inhibition detected. Data shown are the mean of duplicate values with error bars representing SD.

The expected ability of DPP4 to bind to MERS spike was shown by ELISA (Figure 3A), with a K_d_ of 0.06 µg/mL. In a pseudovirus inhibition assay, DPP4 at 20 µg/mL inhibited MERS-CoV pseudovirus entry, with 37% inhibition detected (Figure 3B).

To investigate the binding affinity of DPP4 to SARS-CoV-2 RBD and full-length spike, SPR assays were performed. Experiments were initially performed by immobilising SARS-CoV-2 spike and RBD on a biotin CAPture chip. The positive control, ACE2, was able to bind with SARS-CoV-2 spike with a dissociation constant (K_d_) of 40 nM and with SARS-CoV-2 RBD with a K_d_ of 14 nM (Figure S3). However, non-specific binding of DPP4 to the biotin CAPture chip was detected, resulting in slightly negative sensorgrams (Figure S3). Therefore, the specificity of the binding interaction between DPP4 and SARS-CoV-2 spike and SARS-CoV-2 RBD could not be determined by that method.

**Figure 4:**
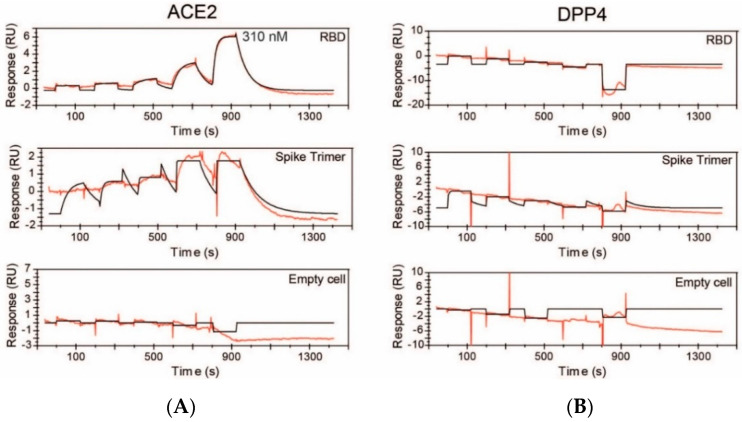
Surface plasmon resonance assays. Purified soluble human ACE2 (**A**) and DPP4 (**B**) were exposed to CM5 chips that had been coated with SARS-CoV-2 RBD or spike protein, or were not coated. Experimental data are shown in red. Calculated data fit using a 1:1 binding model are shown in black. Ligands were injected at increasing concentrations of (**A**) ACE2 at 0.50 nM, 2.5 nM, 12 nM, 62 nM and 310 nM and (**B**) DPP4 at 1.6 nM, 8.0 nM, 40 nM, 200 nM and 1000 nM.

The SPR assay was then performed by immobilising SARS-CoV-2 spike and RBD on a CM5 chip. ACE2 readily bound to RBD with a K_d_ of 100 nM (k_on_ 1.7 × 10^5^ M^−1^ s^−1^; k_off_ 1.7 × 10^−2^ s^−1^), and to spike with a K_d_ of 0.34 nM (k_on_ 3.3 × 10^7^ M^−1^ s^−1^; k_off_ 1.0 × 10^−2^ s^−1^) (Figure 4A). In contrast, no specific binding between DPP4 and either RBD or spike glycoprotein of SARS-CoV-2 was detectable in this assay (Figure 4B). Given the concentrations used, we can infer that any interaction between DPP4 and the spike protein would have a dissociation constant of ~10 μM or weaker.

### 2.3. Protein Structures: Depictions of Binding Surfaces

**Figure 5:**
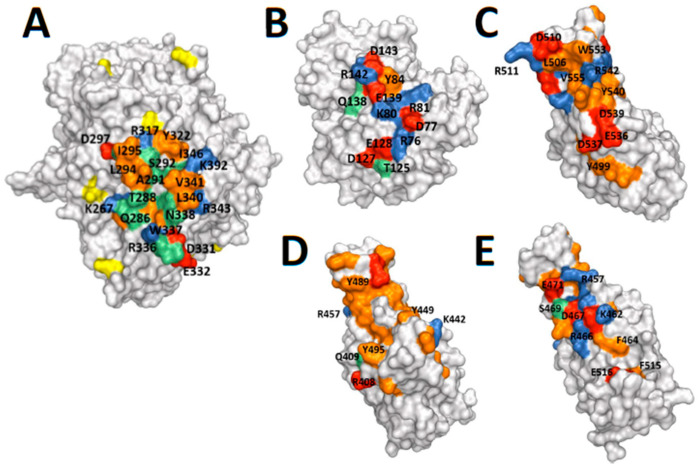
Protein structures. (**A**) DPP4 monomer (PDB ID 1W1I) [46]. (**B**) Adenosine Deaminase (ADA) (PDB ID 1W1I) [46]. (**C**) MERS-CoV spike RBD (PDB ID 4L72) [45], and (**D**,**E**) SARS-CoV-2 spike RBD (PDB ID 6M0J) [48] shown with space-filling surfaces with similar orientation and scale. The observed and predicted interfacial binding sites are highlighted in green, with the charged residues highlighted in red (negative) and blue (positive), and the hydrophobic residues are highlighted in orange. In particular, compared to the other molecules SARS-CoV-2 spike RBD has an extensive hydrophobic surface, and fewer charged residues at the predicted DPP4 binding site that is in a similar location to MERS-CoV spike RBD (**D**) [28], but has more charges surfaces on the opposite side of SARS-CoV-2 spike RBD, which is a potential alternative binding site (**E**) [28]. The *N*-glycosylated residues in DPP4 are highlighted in yellow, and are sufficiently distant from the binding interface to expect that they do not bind to ADA [20].

The protein binding surfaces of the relevant proteins are depicted in Figure 5. The predicted binding sites on DPP4 of ADA, MERS RBD and CoV-2 RBD substantially overlap and are located on blades 4 and 5 of the b-propeller domain of DPP4 [28,45,46]. These three binding sites, of the known ligands and a proposed ligand of DPP4, are similar in their display of some concavity. In contrast, the binding surface on DPP4 is flatter and not complementary in shape. Thus, the topology of each ligand is similar despite very little sequence similarity between ADA and MERS spike. Additionally, there are similarities between how DPP4 binds to MERS RBD and to ADA—in that both interactions are amphiphilic and include a hydrophobic interaction involving Leu294 of DPP4, as well as hydrogen bonding and a salt bridge. The predicted binding site on CoV-2 is somewhat similar to MERS, with predicted van der Waals and hydrogen bonding, but differs in surface charge. Glycans might be important on spike [47]; however, sugars on DPP4 are not in the binding site (Figure 5) so might not be involved on DPP4.

## 3. Discussion

In this study, we developed an optimised purification method for high yields of active pure soluble recombinant human DPP4 with specific activities above 30 U/mg. We showed that purified human DPP4 can bind MERS-CoV spike, but unable to bind either SARS-CoV-2 RBD or SARS-CoV-2 full-length spike. Soluble recombinant active human DPP4 has been purified from insect cells previously using IMAC, with final recoveries of around 20% and comparable specific activities [13,34,35]. The optimised four-step purification strategy developed in this study allowed us to achieve pure soluble human DPP4 from insect cells with recoveries above 20% and yields of up to 1.8 mg and 50 Units per litre of cell culture.

Our novel DPP4 purification procedure included AS precipitation and HIC to remove abundant contaminants that can increase the viscosity of the solution and column pressure in subsequent chromatography steps. A tandem dye affinity chromatography and IMAC approach was employed to further remove impurities with little loss of DPP4 prior to IMAC. This approach minimised the number of chromatographic steps and removed many contaminants prior to IMAC, while minimising time, cost and DPP4 loss, and avoiding size separation that risks removing active oligomeric DPP4. The acidic isoelectric point of DPP4 permits IEX, which was used as a concentration step that maintained final purity above 2000-fold. This pure DPP4 has good stability at 4 °C, as has been observed for natural purified DPP4 [49].

Receptor recognition is an essential early step for coronavirus entry into a cell. The purified soluble human DPP4 protein allowed us to examine its potential interaction with SARS-CoV-2 RBD and full-length spike glycoprotein by SPR. In this study, human ACE2, as an identified entry receptor for SARS-CoV-2, strongly bound to the RBD and full-length spike glycoprotein of SARS-CoV-2. In contrast, DPP4 did not bind in this sensitive assay. Mediation of SARS-CoV-2 entry in non-permissive cells, HeLa and BHK21 that lack human ACE2 has also been shown to be independent of human DPP4 [29,32,33]. These data suggest that human DPP4 is neither an entry receptor nor co-receptor used by SARS-CoV-2. We previously showed that DPP4 from this expression construct is intact and fully active [35,50], and so able to bind to its ligands. Moreover, the enzyme activity of the human DPP4 molecule requires both the *α*/*β*-hydrolase domain and the eight-blade *β*-propeller domain to be intact [43]. Nevertheless, the ability of our DPP4 to bind MERS-CoV spike was verified. Therefore, the inability of our DPP4 to bind SARS-CoV-2 spike is very unlikely to be caused by a defect in the DPP4.

Comparing protein structures provides some understanding of why DPP4 does not bind to CoV-2 RBD. The observed difference in areas of surface charge on SARS-CoV-2 spike compared to the DPP4 binding site on MERS spike offered a potential reason for the observed difference in binding. A limitation of this analysis is that, in nature, proteins can change shape or bind to a different site on the cognate receptor. 

In conclusion, we established and optimised a purification protocol for active recombinant soluble human DPP4 that can yield 1 to 1.8 mg of pure protein per litre of insect cell suspension culture. The availability of large quantities of human soluble DPP4 proteins facilitates further structural studies and substrate and inhibitor discovery to enhance the biochemical understanding of this protease for developing therapeutics for MERS, diabetes, cancer, fibrosis and atherosclerosis.

## 4. Materials and Methods

### 4.1. Materials

*Spodoptera frugiperda 9* (Sf9) insect cells and Cellfectin II Reagent were from Invitrogen (Carlsbad, CA, USA). *Escherichia coli* DH5α cells were from Thermo Fisher Scientific (Waltham, MA, USA). X-Gal was from Bio-Rad (Hercules, CA, USA). Insect-XPRESS medium was from Lonza (Basel, Switzerland). Enzyme substrate H-Gly-Pro-pNA was from Bachem (Bubendorf, Switzerland). Chromatography resins and materials for SPR were from Cytiva (Chicago, IL, USA). SnakeSkin Dialysis Tube, 3.5 kDa molecular weight cut-off (3.5 k MWCO), was purchased from Thermo Fisher Scientific. All other reagents were from Sigma-Aldrich (St. Louis, MO, USA).

### 4.2. Expression and Purification of Active Soluble Human DPP4

#### 4.2.1. Expression of DPP4 in Insect Sf9 Cells

Soluble human DPP4 (residues 29–766; GenBank M80536) was cloned with a *C*-terminal His_6_ –tag into the pMelbac vector and expressed according to the Bac-*N*-Blue baculovirus expression system protocol (Thermo Fisher Scientific) [35]. The expression plasmid construct was transformed into *E. coli* DH5α cells. Positive clones were identified by restriction digest and Sanger Sequencing (The Australian Genome Research Facility; Westmead, NSW, Australia).

Insect Sf9 cells were maintained in Insect-XPRESS medium at 27 °C as either adherent cultures, or as suspension cultures by shaking at 130 rpm. Adherent Sf9 cells (1 × 10^6^ cells/well in a 6-well plate) were transfected with recombinant bacmid DNA using Cellfectin II Reagent. The cells were monitored every 24 h by bright-field microscopy to observe cell lysis. Around 72 h post-transfection, the cell culture supernatant was harvested. Plaque assays were performed to identify positive recombinant stocks. To perform the plaque assay, Sf9 cells (5 × 10^6^ cells/100-mm-plate) were prepared and incubated at 27 °C for 12 to 24 h. Serial dilutions from 10^−1^ to10^−4^ of the transfection viral stock were prepared, and viral dilution at 1 mL was added per plate. The medium was incubated for 1 h at 27 °C and aspirated from the plate. An agarose solution at 5 mL, which included 50 µg/mL X-Gal (5-bromo-4-chloroindol-3-yl β-d-galactopyranoside), was laid over the cells [51]. Plates were then incubated at 27 °C for 7 to 10 days.

Positive recombinant stocks (P0) identified from the plaque assay were used to infect fresh Sf9 cells in 96-well plates (3.6 × 10^4^ cells/well) for screening and selecting the most productive recombinant virus clones [52]. Dilutions of virus to give 5 pfu/well and 0.5 pfu/well were used to infect the Sf9 cells. When cell viability reached <30%, DPP4 expression was measured by enzyme activity assay. Clones with the greatest DPP4 enzyme activity were selected and used to infect fresh Sf9 cells to generate P1 baculovirus stock.

To passage the baculoviral stocks, P1 baculovirus stock was used to infect a fresh 10 mL Sf9 (2 × 10^6^ cells/mL) suspension culture at an MOI of 0.05 to generate P2 baculovirus. Similarly, P2 baculovirus stock was used to infect 500 mL of Sf9 cells to generate high-titre P3 baculovirus working stock. For large scale protein expression, Sf9 suspension cultures were infected with the recombinant P3 baculovirus stock at an MOI of 1 in Insect-XPRESS medium for 7 days. The virus titres of P3 stocks were determined by plaque assay, as described above, using a dilution of 10^−1^ to 10^−7^. The virus titre (pfu/mL) was calculated as (1/dilution) × number of plaques.

#### 4.2.2. Purification of DPP4

Cell culture supernatant was clarified (5375× *g* for 10 min) then solid ammonium sulphate (AS) was added to 35% (*w*/*v*) at 25 °C, and the precipitate was discarded following centrifugation at 25,800× *g* for 30 min at 4 °C. To the supernatant, 80% AS was added, then, following 25,800× *g* for 30 min at 4 °C, the precipitate was retained. The precipitate was solubilised in 10 mM Tris-HCl, pH 7.6, then dialysed against 12% AS in 50 to 100 sample volumes of Buffer A (12% AS in 10 mM Tris-HCl, pH 7.6) overnight at 4 °C. Following centrifugation at 26,000× *g* for 20 min at 4 °C, the supernatant was retained.

Column chromatography used the ÄKTA purifier™ system (Cytiva). A Phenyl Sepharose column (2 × 5 mL) was equilibrated with Buffer A and the flowthrough collected. The bound proteins were eluted with Buffer B (10 mM Tris-HCl, pH 7.6) and discarded. The Phenyl Sepharose flowthrough was dialysed overnight at 4 °C against Buffer C (200 mM NaCl in 10 mM Tris-HCl, pH 7.6). A Blue Sepharose column (1 × 5 mL) was attached upstream of a Nickel Sepharose column and were both equilibrated with 20 mM imidazole in Buffer C. Imidazole at 20 mM was added to the dialysed sample, which was then applied to these columns. DPP4 was eluted from the Nickel Sepharose (1 × 5 mL) column with 100 mM imidazole in Buffer C. The eluted fractions were dialysed overnight against Buffer B and then applied to a DEAE Sepharose column (2 × 1 mL) that had been pre-equilibrated with Buffer B. DPP4 was eluted from the DEAE column with Buffer C. The purified proteins were stored in Buffer C with 1 mM EDTA and 10% glycerol at −80 °C. Glycerol is necessary as a cryopreservative, and EDTA prevents inhibition of DPP4 by metal ions [53].

### 4.3. Expression and Purification of SARS-CoV-2 Full-Length Spike, SARS-CoV-2 RBD, and Human ACE2

#### 4.3.1. Generation of Expression Constructs

The expression plasmid for soluble trimeric SARS-CoV-2 spike protein (residues 1–1208) was generously provided by Dr Florian Krammer (Icahn School of Medicine, Mt Sinai) [54,55]. The SARS-CoV-2 spike expression construct includes the proteins native signal peptide (residues 1–14) to enable secretion, proline substitutions at residues 986 and 987 for stability, a GSAS substitution at the furin cleavage site (residues 682–685), and an *N*-terminal His_6_-tag to allow purification. The SARS-CoV-2 receptor-binding domain (RBD) of the spike protein (residues 328–531) was cloned into the pCAGGS expression plasmid with an *N*-terminal IgK signal peptide, to target the protein for secretion, and a C-terminal His_9_-tag and Avitag™ to enable purification. The soluble domain of the human ACE2 receptor (residues 1–614) was cloned into the pcDNA3.1 expression plasmid with a *C*-terminal His_9_-tag and Avitag™ to enable purification. The native signal peptide of ACE2 (residues 1–18) was included to allow secretion of the protein upon expression. To allow specific enzymatic biotinylation of proteins possessing an Avitag™, full-length *E. coli* biotin ligase BirA was cloned into pcDNA3.1 with an *N*-terminal Cd4 signal peptide, to enable secretion of the protein, and no tag for affinity purification.

#### 4.3.2. Expression and Purification of SARS-CoV-2 Full-Length Spike, SARS-CoV-2 RBD, and Human ACE2

SARS-CoV-2 full-length spike, SARS-CoV-2 RBD, and human ACE2 were expressed in EXPI293F™ cells at 37 °C using transient transfection with 25 kDa linear polyethyleneimine (PEI). EXPI293F™ cells were transfected at a cell density of 3 × 10^6^ cells/mL with pre-formed DNA-PEI complexes (2 µg/mL DNA and 8 µg/mL PEI), and cultures were harvested 72 h post-transfection by centrifugation at 4000× *g* for 20 min. Supernatants from the centrifugation were supplemented with 20 mM HEPES pH 8.0 and were passed over Ni-NTA agarose equilibrated with a buffer comprising 20 mM NaH_2_PO_4_ pH 8.0, 500 mM NaCl, and 20 mM imidazole for purification via His-tag affinity chromatography. Proteins were eluted from the Ni-NTA agarose using a buffer containing 20 mM NaH_2_PO_4_ pH 8.0, 300 mM NaCl, and 500 mM imidazole. Eluates from Ni-NTA purification were concentrated and further purified using a Superdex 200 10/30 GL column in a buffer comprising of 20 mM HEPES pH 7.5 and 150 mM NaCl.

For SPR, SARS-CoV-2 spike was chemically biotinylated at the *N*-terminus using EZ-link™ NHS-Biotin and performing the biotinylation reaction overnight at pH 6.5 and 4 °C. SARS-CoV-2 RBD was enzymatically biotinylated at the *C*-terminal Avitag™ by co-transfecting RBD with a BirA expression construct and supplementing the culture media with 100 µM biotin during expression.

### 4.4. SDS–PAGE

Protein concentration was measured by Bradford Protein Assay Kit (Pierce, Waltham, MA, USA). Standards used Bovine Serum Albumin (BSA). Absorbance was read at 595 nm. Protein samples were diluted in NuPAGE Sample Reducing Agent (10X) and NuPAGE LDS Sample Buffer (4×) and boiled for 5 min before loading on 4–12% Bis–Tris NuPAGE gradient gel (Thermo Scientific, Waltham, MA, USA). Proteins were stained with Sypro ruby or Colloidal blue (Thermo Scientific) for visualisation. Molecular masses were estimated by comparison with a Page Ruler Prestained Protein Ladder (Thermo Scientific).

### 4.5. Enzyme Assays

Enzyme activity was measured as previously described [56] and detailed in Supplementary Material A. Hydrolysis of the DPP4 substrate H-Gly-Pro-pNA was measured by absorbance at 405 nm, with 570 nm for background subtraction, each 30 s for 10 min at 37 °C. A unit (U) of activity is defined as an enzyme activity that hydrolyses 1.0 μmol of substrate per minute at 37 °C.

### 4.6. Surface Plasmon Resonance Assay

SPR was performed using a BIAcore T200 instrument (Cytiva) and conditions similar to methods described for investigating DPP4 and ACE2 binding with the spike and RBD of MERS [26,27]. Biotinylated SARS-CoV-2 spike and SARS-CoV-2 RBD were immobilised onto either a CM5 chip (Cytiva) via amine coupling or a biotin CAPture chip (Cytiva). Both spike and RBD proteins of SARS-CoV-2 were immobilised on the CM5 chip at about 500 response units. SARS-CoV-2 spike was immobilised on the biotin CAPture chip at ~500 response units, and SARS-CoV-2 RBD was immobilised on the biotin CAPture chip at ~50 response units. Single-cycle kinetic experiments for the binding of ACE2 (0.50 nM, 2.5 nM, 12 nM, 62 nM and 310 nM) and human soluble DPP4 (0, 1.6 nM, 8.0 nM, 40 nM, 200 nM and 1000 nM) were performed at 25 °C using an HBS-EP buffer consisting of 10 mM HEPES, pH 7.5, 150 mM NaCl, 3 mM EDTA and 0.01% (*v*/*v*) Tween-20 as the running buffer. The sensor surface was regenerated using an injection of 5 mM NaOH between each cycle when required. Binding kinetics were analysed with the software BIAevaluation Version 3.1 using the 1:1 Langmuir binding model.

### 4.7. ELISA

Binding of recombinant DPP4 to MERS-CoV spike clamp protein was determined via ELISA. Recombinant DPP4 was diluted to 2 µg/mL in PBS, and 50 µL was coated on a Nunc MaxiSorp™ high protein-binding capacity 96 well ELISA plate overnight at 4 °C. Plates were blocked with 150 µL/well of 5% KPL Milk Dilutent/Blocking solution concentrate (SeraCare, Milford, MA, USA) in PBS with 0.05% Tween-20 for 30 min at room temperature. Next, serial dilutions of MERS-CoV or SARS-CoV-2 spike protein, or a control protein, were added and incubated for 1 h at 37 °C. Plates were then washed three times with PBS with 0.05% Tween-20 before incubation with 2 µg/mL of an antibody towards the clamp domain, HIV1281 [57]. Plates were washed as before and incubated with a goat anti-human HRP secondary antibody (1:2000 dilution, Sigma Aldrich) for 1 h at 37 °C. Plates were washed a final time before the binding was revealed by the addition of tetramethylbenzidine (TMB) solution (Life Technologies) for 5 min. Reactions were stopped by the addition of 1 M sulfuric, acid and absorbance read at 450 nm. Absorbance was plotted using Graphpad Prism software version 8 using a one-site specific binding model.

### 4.8. MERS-CoV Pseudovirus Assay

MERS-CoV pseudovirus was made as previously described [58]. Briefly, HEK293T cells were seeded in DMEM 10% FCS (D10) media (Invitrogen) at a density of 2 × 10^6^ cells in a 10 cm^2^ dish and incubated at 37 °C 5% CO_2_. The following day, cells were transfected with 1 µg of MERS-CoV full-length spike (residues 1–1353, GenBank: AHX00711.1), constructed using a gBlock (Integrated DNA Technologies, Coralville, IO, USA), the CMV promoter and infusion cloning (TakaraBio, Mountain View, CA, USA) in pNBF, along with 1 µg p8.91 (encoding HIV-1 gag-pol) and 1.5 µg pCSFLW [58] (encoding firefly luciferase reporter lentivirus backbone) with 14 µL Lipofectamine LTX and 3.5 µL PLUS reagent (Invitrogen) and incubated at 37 °C 5% CO_2_. The next day, the transfection mix was removed and replaced with 7 mL D10 media and incubated for a further 24 hrs. The virus was then harvested, and media was replaced for additional harvests 12 and 24 hrs later. Pooled harvests were centrifuged at 4 °C for 10 min at 1300× *g* to remove cellular debris. To measure MERS-CoV pseudovirus titer, target Huh-7 cells (Japanese Collection of Research Bioresources) were plated at a density of 2 × 10^4^ cells/well of a white Nunc MicroWell^TM^ 96-well plate in D10 media and incubated at 37 °C 5% CO_2_. The next day, MERS-CoV pseudovirus was titrated on target cells 5-fold in D10 media and incubated at 37 °C 5% CO_2_. After 72 hrs, firefly luciferase reporter activity was measured by discarding supernatant and adding 50 µL/well of a 1:1 mix of Bio-Glo Luciferase Assay System (Promega) and serum-free DMEM. The plate was incubated for 10 min at room temperature before reading on a Varioskan LUX (ThermoFisher).

To measure virus inhibition, target Huh-7 cells were plated at a density of 2 × 10^4^ cells per well of white Nunc MicroWell^TM^ 96-well plate in D10 media and incubated at 37 °C 5% CO_2_. The next day, inhibitors DPP4, ACE2 and Mab m336 [59], and non-specific mAb control C05 [57], were each diluted to an appropriate concentration in D10 media and incubated with MERS-CoV pseudovirus, at a dilution that would yield ~2 × 10^6^ RLU, for 1 hr at 37 °C 5% CO_2_. Inhibitor/virus mixture was then added on to Huh-7 cells and incubated for a further 72 hrs at 37 °C 5% CO_2_. Firefly luciferase reporter activity was quantified for virus titration.

### 4.9. Protein Structure Depictions

Structures of SARS-CoV-2 spike RBD [48], and human DPP4 complexed with MERS-CoV spike receptor-binding domain [45] and with human ADA [46] are available in the PDB, with access codes 6M0J, 4L72 and 1W1I, respectively. Protein structures were visualised and images generated using PyMOL software (Schrodinger LLC.; version 2.4.1; New York, NY, USA).

## Figures and Tables

**Table 1:** Representative purification of DPP4 from Sf9 cells.

	Volume (mL)	Protein (mg/mL)	Total Protein (mg)	Total Activity (U)	Specific Activity (U/mg)	Fold-Purification *	Yield (%) **	Step-Wise DPP4 Loss (%) #	Step-Wise Total Protein Loss (%) ##
Cell culture supernatant	1000	7.3	7300	144	0.020	1	100	0	0
35% AS	1046	3.9	4079	109	0.027	1.4	76	24	44
80% AS	82	7.2	589	85	0.14	7.0	59	22	86
Phenyl Sepharose	93	4.1	381	77	0.20	10	53	9.4	35
Ni Sepharose	41	0.034	1.4	64	46	2300	44	16	99.6
DEAE Sepharose	4.4	0.23	1.0	42	42	2100	29	35	29

* Fold-purification is specific activity after the step/specific activity of the starting material (cell culture supernatant). ** Yield is Units of DPP4 as a % of DPP4 Units in the starting material. # Step-wise DPP4 loss (%) is 100 × (total activity after the previous step − total activity after this step)/total activity after the previous step. ## Step-wise total protein loss (%) is 100 × (total protein after previous step − total protein after this step)/total protein after the previous step.

**Table 2:** DPP4 purification yields and specific activity from five replicate experiments.

Purification Replicate	Culture Supernatant (L)	Total Protein (mg)	Enzyme Activity (U/mL)	Specific Activity (U/mg)	Final Yield (mg/L Culture)
1	1	1.2	45	37	1.2
2	1.1	1.2	52	43	1.1
3	0.98	1.5	44	29	1.6
4	0.98	1.8	61	34	1.9
5	0.99	1.0	42	40	1.0
Mean ± SD	1.0 ± 0.1	1.3 ± 0.3	49 ± 7.8	36 ± 5.4	1.4 ± 0.4

## Supplementary Materials

The following are available online. Figure S1: Precipitation of soluble DPP4 from culture supernatant at various saturations levels of ammonium sulphate; Figure S2: Purified DPP4 on SDS–page; Figure S3: Surface plasmon resonance assay characterising binding between DPP4 and SARS-CoV-2 RBD and spike with a biotin CAPture chip; Supplementary Material A: Methods of assay and storage for purified recombinant soluble human DPP4 protein.

## Abbreviations

Adenosine deaminase binding protein (ADAbp), diethylaminoethyl (DEAE), dipeptidyl peptidase 4 (DPP4), type 2 diabetes mellitus (T2DM), glucagon-like peptide-1 (GLP-1), gastric inhibitory polypeptide (GIP), angiotensin-converting enzyme 2 (ACE2), receptor-binding domain (RBD), severe acute respiratory syndrome (SARS), middle east respiratory syndrome (MERS), surface plasmon resonance (SPR), ammonium sulphate (AS), hydrophobic interaction chromatography (HIC), immobilised metal affinity chromatography (IMAC), ion-exchange chromatography (IEX), *Spodoptera frugiperda 9* (Sf9), multiplicity of infection (MOI), polyethyleneimine (PEI).
