# Supplementary material for: A Novel Purification Procedure for Active Recombinant Human DPP4 and the Inability of DPP4 to Bind SARS-CoV-2
Source: Molecules. 2020 Nov 18;25(22):5392. doi: 10.3390/molecules25225392 (PMC7698748; doi:10.3390/molecules25225392)
Supplement: Supplementary file 1 [file molecules-25-05392-s001.pdf]

Supplementary information

A novel purification procedure for active recombinant human DPP4 and the inability of DPP4 to bind SARS_CoV_2

Cecy R Xi, Arianna Di Fazio, Naveed Ahmed Nadvi, Karishma Patel, Michelle Sui Wen Xiang, Hui Emma Zhang, Chandrika Deshpande, Jason K K Low, Xiaonan Trixie Wang, Yiqian Chen, Christopher L D McMillan, Ariel Isaacs, Brenna Osborne, Ana Júlia Vieira de Ribeiro, Geoffrey W McCaughan, Joel P Mackay, W Bret Church, Mark D Gorrell

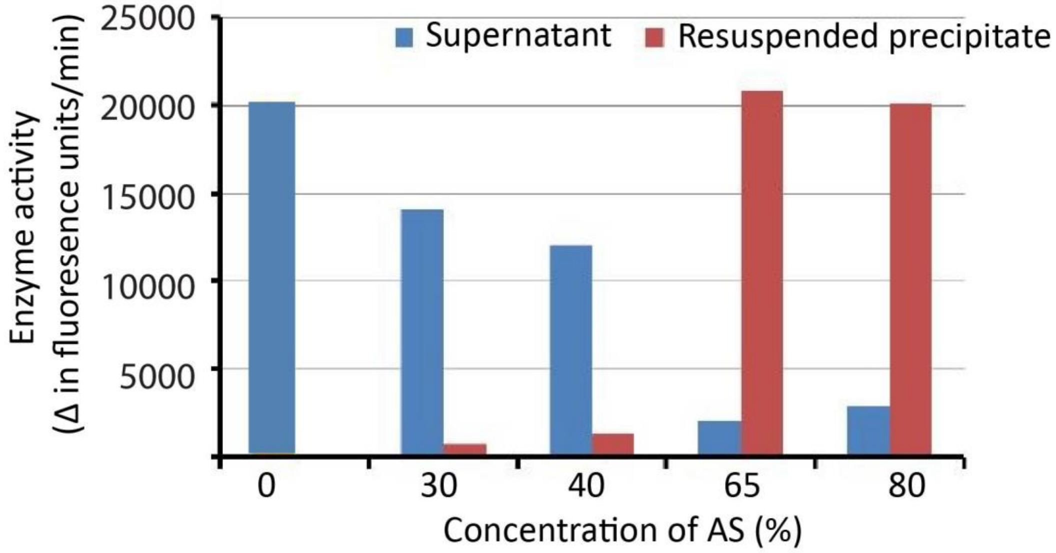

Figure S1: Precipitation of soluble DPP4 from culture supernatant at various saturation levels of ammonium sulfate. Solid ammonium sulfate (AS) at 0%, 35%, 40%, 65% and 80% (w/v) was added to Sf9 cell culture supernatant. DPP4 enzyme activity was measured in the supernatant and re-suspended precipitate.

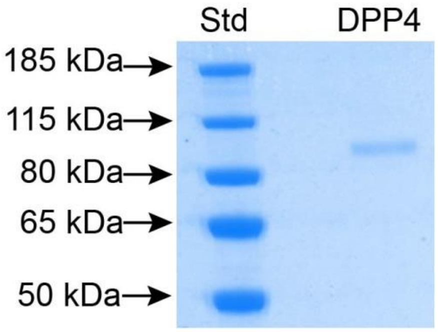

Figure S2: Purified DPP4 on SDS-PAGE. Purified DPP4 was detected at 90 kDa with Colloidal blue stain.

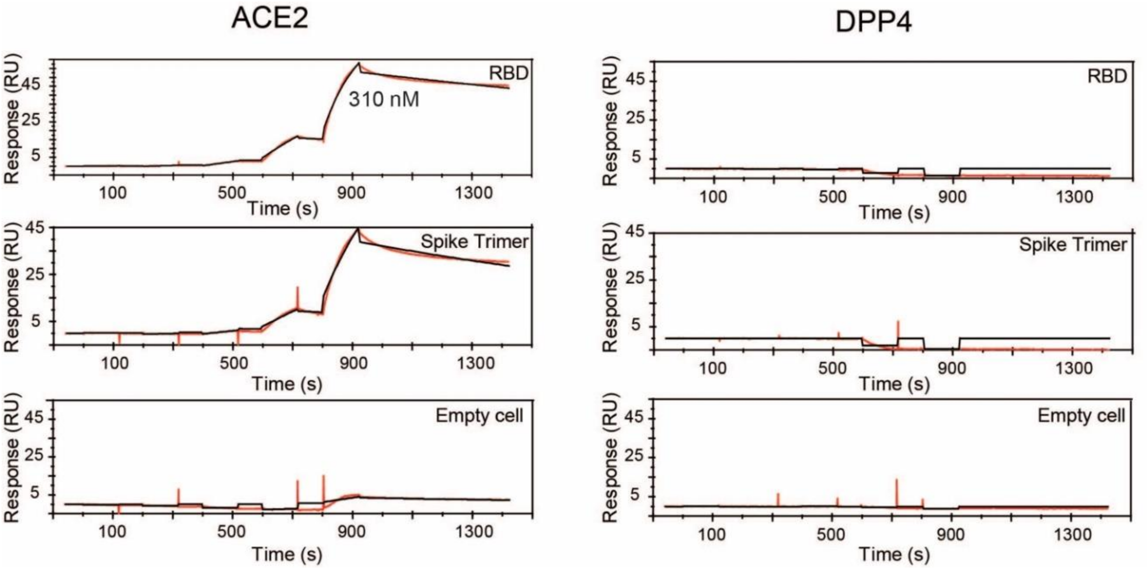

ACE2	K_d	K_{on}	K_{off}
RBD	1.4×10^{-8}	2.8×10^{4}	3.8×10^{-4}
Spike	4.0×10^{-8}	1.5×10^{4}	6.0×10^{-4}

Figure S3. **Surface plasmon resonance assay characterising binding between DPP4 and SARS-CoV-2 RBD and spike with a biotin CAPture chip**. Experimental data are shown in red. Calculated fits using a 1:1 binding model are shown in black. Gradient concentrations of (A) ACE2 at ACE2 at 0.50 nM, 2.5 nM, 12 nM, 62 nM and 310 nM and (B) DPP4 at 1.6 nM, 8.0 nM, 40 nM, 200 nM and 1000 nM were flowed over the chip surface bound with RBD and spike glycoprotein.

SUPPLEMENTARY MATERIAL A: METHODS OF ASSAY AND STORAGE FOR PURIFIED RECOMBINANT SOLUBLE HUMAN DPP-IV PROTEIN.

Dipeptidyl peptidase IV, EC#: 3.4.14.5, is also known as DPIV, DPP-IV, DPP4, adenosine deaminase binding protein (ADAbp), thymocyte activation molecule (THAM).

This is an active soluble form of human DPP-IV, residues 29-766. Six histidines were added at the C-terminus.

Molecular Weight: approximately 90,000 daltons [39]

Source:

The cDNA, Genbank ID M80536, was from human lymphocytes [5]. The expression construct was made by PCR from human liver RNA and expressed in a baculovirus / Sf9 insect cell system following the methods described by [35,39].

Unit Definition:

One unit will produce 1.0 µmole of p-nitroaniline from Gly-Pro p-nitroanilide per min in reaction buffer: 100 mM Tris-HCl at pH 7.6. All reagents were prepared in this reaction buffer.

The extinction co-efficient of pNA at 405 nm was 6.993 mM^{-1} cm^{-1}. This was calculated from a pNA titration.

Assay in 96-well microplate [50,56]

295 µl H-Gly-Pro p-nitroanilide (Bachem: L-1880, or Sigma-Aldrich: G0513, at 1 mM) were added to 5 µl DPP-IV sample (enzyme) in each well and the reaction was incubated at 37 degrees Celsius. The OD was monitored continuously at 405 nm from time zero until 10 minutes or until the OD exceeded 1.0. Enzyme activity was converted to the change in absorbance units per minute. OD (405 nm) was measured using a FLUOstar Omega microplate reader (BMG LABTECH). Enzyme activity (Units/mL) was calculated using an extinction coefficient of

6.993 in the formula:

[total vol of 300 µl x change in OD / minutes] / [6.993 x 2.5 µl sample volume].

Authentic pNA (Sigma cat# 185310) was used to calculate the extinction coefficient at 405 nm from a standard curve. pNA standard solutions (300 µl) were prepared in reaction buffer.

Assay in cuvette:

0.42 ml GlyPro p-nitroanilide (Bachem: L-1880 or Sigma: G0513)(1 mM) was added to 0.005 ml of DPP-IV sample (enzyme) being tested in a 0.5 ml cuvette. The reaction was incubated at ambient temperature and the OD monitored continuously at 405 nm from time zero until 10 minutes or until the OD exceeds 1. The change in absorbance units per minute was calculated. OD (405 nm) was measured using a spectrophotometer. Enzyme activity (Units/ml) was calculated using the calculated extinction coefficient (E) in the formula:

[total vol of 0.425 ml x change in OD / minutes] / [E x 0.005 ml sample volume].

References

5. Tanaka, T.; Duke-Cohan, J. S.; Kameoka, J.; Yaron, A.; Lee, I.; Schlossman, S. F.; Morimoto, C. Enhancement of antigen-induced T-cell proliferation by soluble CD26/dipeptidyl peptidase IV. *Proc. Natl Acad. of Sci.* **1994**, *91*, 3082-3086.

35. Park, J.; Knott, H.M.; Nadvi, N.A.; Collyer, C.A.; Wang, X.M.; Church, W.B.; Gorrell, M.D. Reversible inactivation of human dipeptidyl peptidases 8 and 9 by oxidation. *Open Enzym. Inhib. J.* **2008**, *1*, 52–61, doi:10.2174/1874940200801010052.
39. Ajami, K.; Pitman, M. R.; Wilson, C. H.; Park, J.; Menz, R. I.; Starr, A. E.; . . . Gorrell, M. D. Stromal cell-derived factors 1 alpha and 1 beta, inflammatory protein-10 and interferon-inducible T cell chemo-attractant are novel substrates of dipeptidyl peptidase 8. *FEBS Lett.* **2008** *582*, 819-825. doi:10.1016/j.febslet.2008.02.005
50. Sinnathurai, P.; Lau, W.; Vieira de Ribeiro, A.J.; Bachovchin, W.W.; Englert, H.; Howe, G.; Gorrell, M.D. Circulating fibroblast activation protein and dipeptidyl peptidase 4 in rheumatoid arthritis and systemic sclerosis. *Int. J.* Rheum. Dis. **2018**, *21*, 1915–1923, doi:10.1111/1756-185X.13031
56. Keane, F.M.; Yao, T.-W.; Seelk, S.; Gall, M.G.; Chowdhury, S.; Poplawski, S.E.; Gorrell, M.D. Quantitation of fibroblast activation protein (FAP)-specific protease activity in mouse, baboon and human fluids and organs. *FEBS Open Bio.* **2014**, *4*, 43–54, doi:org/10.1016/j.fob.2013.12.001
